# Sub-Cellular Localisation Studies May Spuriously Detect the Yes-Associated Protein, YAP, in Nucleoli Leading to Potentially Invalid Conclusions of Its Function

**DOI:** 10.1371/journal.pone.0114813

**Published:** 2015-02-06

**Authors:** Megan L. Finch, Adam M. Passman, Robyn P. Strauss, George C. Yeoh, Bernard A. Callus

**Affiliations:** 1 School of Chemistry and Biochemistry, University of Western Australia, Crawley, Western Australia, 6009, Australia; 2 Harry Perkins Institute of Medical Research, QEII Medical Centre, Nedlands and Centre for Medical Research, the University of Western Australia, Crawley, Western Australia, 6009, Australia; University of Navarra School of Medicine and Center for Applied Medical Research (CIMA), SPAIN

## Abstract

The Yes-associated protein (YAP) is a potent transcriptional co-activator that functions as a nuclear effector of the Hippo signaling pathway. YAP is oncogenic and its activity is linked to its cellular abundance and nuclear localisation. Activation of the Hippo pathway restricts YAP nuclear entry via its phosphorylation by Lats kinases and consequent cytoplasmic retention bound to 14-3-3 proteins. We examined YAP expression in liver progenitor cells (LPCs) and surprisingly found that transformed LPCs did not show an increase in YAP abundance compared to the non-transformed LPCs from which they were derived. We then sought to ascertain whether nuclear YAP was more abundant in transformed LPCs. We used an antibody that we confirmed was specific for YAP by immunoblotting to determine YAP’s sub-cellular localisation by immunofluorescence. This antibody showed diffuse staining for YAP within the cytosol and nuclei, but, noticeably, it showed intense staining of the nucleoli of LPCs. This staining was non-specific, as shRNA treatment of cells abolished YAP expression to undetectable levels by Western blot yet the nucleolar staining remained. Similar spurious YAP nucleolar staining was also seen in mouse embryonic fibroblasts and mouse liver tissue, indicating that this antibody is unsuitable for immunological applications to determine YAP sub-cellular localisation in mouse cells or tissues. Interestingly nucleolar staining was not evident in D645 cells suggesting the antibody may be suitable for use in human cells. Given the large body of published work on YAP in recent years, many of which utilise this antibody, this study raises concerns regarding its use for determining sub-cellular localisation. From a broader perspective, it serves as a timely reminder of the need to perform appropriate controls to ensure the validity of published data.

## Introduction

The Yes-associated protein (YAP) is a potent oncogene and functions as a transcriptional co-activator that can interact with a variety of DNA-binding transcription factors in the nucleus to activate target gene expression [1–5]. YAP’s oncogenic activity is linked to its cellular abundance. Consistent with this, amplification of the YAP gene has been observed in several cancer types including breast [6], medulloblastoma [7], hepatocellular (HCC) [8], and squamous cell carcinomas [9]. Increased YAP abundance is also seen in liver [10, 11], breast [12], prostate [11] and colorectal [13] cancers, squamous cell [14], lung and colon adenocarcinomas, and ovarian carcinomas [12]. Over-expression of YAP in the liver of transgenic mice results in a 4–5 fold increase in liver size and can lead to the formation of HCC-like tumors [13, 15]. Lastly, YAP abundance was shown to be an independent prognostic marker for overall survival and disease-free survival of HCC patients [10].

YAP activity is also dependent on its sub-cellular localisation, and is regulated by shuttling between the cytoplasm and nucleus. Interaction between the PDZ-domain containing protein, ZO-2, and YAP’s C-terminal PDZ-binding motif appears necessary for its nuclear localisation [16, 17]. Additionally, the cytoplasmic localisation of YAP is regulated by several factors and is principally controlled by the Hippo pathway [15, 18–20]. Cell-cell contact is one mechanism that activates the Hippo signaling cascade resulting in activation of the Lats1/2 kinases that phosphorylate YAP on multiple serine residues [11, 21]. Phosphorylated YAP remains sequestered in the cytoplasm bound to 14-3-3 proteins and is eventually degraded following additional phosphorylation by CK1 or GSK-3 kinases [11, 19, 22]. Similarly, phosphorylation of YAP by Akt results in its retention in the cytoplasm bound to 14-3-3 proteins [18].

Other mechanisms for attenuating YAP function have also been reported. For example, direct interaction with α-catenin promoted YAP cytoplasmic localisation and loss of YAP function in the nucleus [23]. Similarly association with angiomotin promotes YAP localisation to cytoplasmic/tight junctions thereby reducing its nuclear activity [24, 25]. More recently, Oudoff et al. reported a novel mechanism of localisation control whereby Set7-mediated methylation of YAP resulted in its cytoplasmic retention [26]. Precisely how YAP methylation influences its localisation is unclear. The authors proposed that since the site of lysine methylation is proximal to the PDZ-binding motif, methylation of this lysine residue may disrupt the interaction between YAP and ZO-2, impeding its nuclear translocation.

Numerous publications have reported increased YAP abundance and nuclear localisation in tumorigenesis [8, 10–12, 15, 27]. Previously, we reported that YAP abundance is increased in tumorigenic compared to non-tumorigenic liver progenitor cells (LPCs) [28]. This is consistent with reports that YAP overexpression promotes tumorigenic characteristics including growth in low serum and anchorage-independent growth [6, 22], a feature of LPCs that have undergone tumorigenic transformation [28, 29]. Whether its sub-cellular localisation further contributes to differences in YAP activity in non-tumorigenic compared to tumorigenic LPCs is unknown. We hypothesized that tumorigenic LPCs would show an increased proportion of nuclear YAP compared to non-tumorigenic LPCs. To test this we employed immunofluorescence with a widely used antibody to determine YAP’s sub-cellular localisation in transformed and non-transformed LPCs. We find that YAP spuriously localised to the nucleoli of LPCs and this staining was non-specific. This finding emphasizes the need to perform appropriate controls to confirm intracellular staining patterns; in this instance with respect to YAP. In their absence, the validity of conclusions based on such data is open to question.

## Materials and Methods

### Antibodies and chemicals

Anti-YAP (#4912) was purchased from Cell Signaling Technology (CST) (Genesearch, Arundel, QLD). Anti-fibrillarin (#ab4566) was purchased from Abcam (Sapphire Biosciences, Waterloo, NSW). Anti-β-actin (#A1978) and 4-hydroxytamoxifen (#H7904) were purchased from Sigma-Aldrich (Castle Hill, NSW).

### Plasmids and cDNAs

Expression plasmids containing the human YAP isoforms, hYAP1-1β (hYAP1) and hYAP1-2α (hYAP2), and a partial cDNA clone (clone ID mYAP6) containing the full coding region corresponding to mouse YAP (mYAP) (NCBI NM_009534.3) were generously provided by Marius Sudol (Geisinger Clinic, Danville, PA). mYAP∆TD was generated by polymerase chain reaction using the primers 5’GTAGGATCCATGGAGCCCGCGCAACA and 5’GTGTCTAGACTATGGGCTCTGGGGAGCCAA to introduce a stop codon at position 276 of mYAP. mYAP, mYAP∆TD, hYAP1 and hYAP2 cDNAs were sub-cloned into the 4-hydroxytamoxifen (4HT) inducible lentiviral vector, pF-5xUAS-MCS-W-SV40puro [30], using unique BamHI and Xba1 restriction sites. All constructs were verified by sequencing. Lentiviral YAP (TRCN0000238432) and control (SHC202) shRNA plasmids (pLKO.1-puro) were purchased from Sigma-Aldrich.

### Cell Culture

BMOL 1 and BMOL-TAT LPCs have been described previously [31]. BMEL Actin-EGFP (BMEL A-EGFP) and BMOL 2 were generated using the method described by Strick-Marchand et al. [32]. Written consent to obtain mouse liver tissue from wild-type mice to generate the BMOL 2 LPC line and from embryonic liver from Actin-EGFP transgenic mice was to generate the BMEL A-EGFP LPC line was obtained from the Animal Ethics Committee at the University of Western Australia (Protocol: RA/3/100/839) prior to the commencement of mouse work. LPCs were maintained in Williams’ E Medium (Sigma, #W4125-10X1L) supplemented with 5% (v/v) fetal bovine serum (Fisher Biotec, WA #FBS-001-AU), 2.5 μg/mL amphotericin B (Life Technologies, Mulgrave, VIC #15290-018), 80 U/mL penicillin G/675 μg/mL streptomycin, 2 mM L-glutamine, 10 μg/mL Humulin R U-100 (UWA Pharmacy, Crawley, WA), 30 ng/mL insulin-like growth factor II (ProSpec, Ness-Ziona, Israel #CYT-265) and 20 ng/mL epidermal growth factor (BD Biosciences, North Ryde, NSW #354001) in a humidified atmosphere of 5% CO_2_ at 37°C. Wild-type MEFs [33], NIH3T3 [34], D645 [35] and 293T [36] cells were provided by David Vaux (WEHI) and grown continuously in Dulbecco’s Modified Eagle medium (Life Technologies, #11885) supplemented with 10% (v/v) fetal bovine serum, 80 U/mL penicillin G/675 μg/mL streptomycin, 2 mM L-glutamine in a humidified atmosphere of 10% CO_2_ at 37°C.

Lentiviruses were prepared by transfecting 293T cells with the appropriate lentiviral vector together with pCMV-∆R8 and pVSV-G packaging constructs using Effectene as described previously [33]. After 48 h viral supernatants were filtered, mixed with polybrene (4 μg/mL), and added to target cells which were then centrifuged at 2 500g for 90 min at room temperature. Stable shRNA knockdown cells were selected in the presence of puromycin (Sigma, #P7255). Stably infected 4HT-inducible cell lines were selected in the presence of puromycin and hygromycin B (Roche, #10843555001) as described before [33]. Gene expression in target cells was induced by the addition of 100 nM 4HT.

### Cell lysis and immunoblotting

Cells were harvested and washed once with phosphate buffered saline (PBS) then resuspended in DISC lysis buffer [37] supplemented with 1X complete protease inhibitor cocktail (Roche), 10 mM sodium fluoride, 2 mM sodium pyrophosphate, 1 mM sodium molybdate and 5 mM ß-glycerophosphate and incubated for 30 min on ice and centrifuged at 16 000g for 10 min at 4°C. Clarified lysates (50 μg) were mixed with sample buffer, boiled and separated by sodium dodecyl sulphate polyacrylamide gel electrophoresis (SDS-PAGE) on Tris-glycine gels then transferred to Hybond C membrane (GE, Castle Hill, NSW). Membranes were blocked in 5% skim milk powder in Tris-buffered saline containing 0.1% Tween-20 (TBST) and incubated with primary antibody. Membranes were washed with TBST, incubated with horseradish peroxidase-conjugated secondary antibody (GE) and washed with TBST before detection with enhanced chemiluminescence (Millipore).

### Immunofluorescence

MEFs and LPCs were seeded into 60 mm dishes containing 13 mm, collagen (Sigma, #C3867-1VL)-coated coverslips (Hurst Scientific, WA). After 24 h cells were fixed by incubation with PBS containing 4% (w/v) paraformaldehyde (Merck, #104005100) and 4% (w/v) sucrose (Merck, #10274) for 15 min at room temperature. After washing twice with PBS for 5 min, fixed cells were blocked and permeabilized by incubation with PBS containing 5% (w/v) bovine serum albumin (BSA) (Sigma, #A7906) and 0.3% (v/v) Triton X-100 (Sigma, Cat. T-9284) for 1 h at room temperature.

Primary antibodies were diluted in 1% (w/v) BSA/0.3% (v/v) Triton X-100/PBS and applied to coverslips and incubated at 4°C overnight. After three washes with PBS, coverslips were incubated with secondary antibody diluted in 1% (w/v) BSA/0.3% (v/v) Triton X-100/PBS at room temperature for 1 h. Cells were washed twice with PBS, before 0.3 μM Hoechst stain diluted in PBS was applied for 5 min to stain nuclei. Cells were washed a further three times before coverslips were mounted onto glass slides using Gelvatol mounting medium (10.5% (w/v) polyvinyl alcohol, 21% (v/v) glycerol, 0.106 M Tris pH 8.5, sodium azide). Slides were viewed using an Olympus IX71 microscope fitted with a U-RFL-T fluorescent lamp, and images were captured using DP Controller software (Olympus Corporation, 2.1.1.183).

D645 cells were grown overnight on coverslips with or without 100 nM 4HT. Cells were fixed in 3.2% paraformaldehyde in PBS for 20 min at room temperature and washed three times with PBS then incubated in PBS containing 150 mM glycine for 15 min. Cells were permeabilised by incubation with 0.5% Triton X-100 in PBS for 5 min and washed twice with PBS. Cells were blocked by the addition of 1% (w/v) BSA/PBS for 30 min before primary antibody diluted in 1% (w/v) BSA/PBS was added for 1 h at room temperature. After four washes with PBS the secondary antibody diluted in 1% (w/v) BSA/PBS was applied to the cells for 1 h at room temperature. Cells were washed three times with PBS before a final wash with PBS containing 1 μM Hoechst dye to stain nuclei then mounted onto glass slides and viewed as described above.

### MitoTracker and immunofluorescence

MEFs stably infected with control shRNA were labelled with 100 nM MitoTracker Red CMXRos (CST, Cat. 9082) for 15 min at 37°C. Cells were then washed and trypsinized, before being mixed 1:1 with unlabelled MEFs stably infected with YAP shRNA and seeded into 60 mm dishes containing 13 mm coverslips. Cells were allowed to settle for 4 h before cells were fixed and stained for YAP as described above.

The protocol for staining mouse liver tissue is provided in S1 Methods.

### Ethics statement

All animal procedures were performed in accordance with guidelines specified by the Animal Ethics Committee of The University of Western Australia (approved protocols RA/3/100/1224 and RA/3/100/839) and the National Health and Medical Research Council of Australia.

## Results

We showed previously that YAP abundance is increased in tumorigenic compared with non-tumorigenic LPCs [28]. However, in this instance the tumorigenic cells were not derived from the non-tumorigenic cells but represented independent cell lines. To resolve this, we sought to ascertain YAP abundance in pairs of LPCs where the transformed LPCs were derived from their non-transformed counterparts. Since cellular YAP activity is dependent on both the amount of protein as well as its localisation within the cell, it was necessary to establish these parameters in both non-transformed and transformed LPCs to ascertain its role in tumorigenesis.

**Figure 1:**
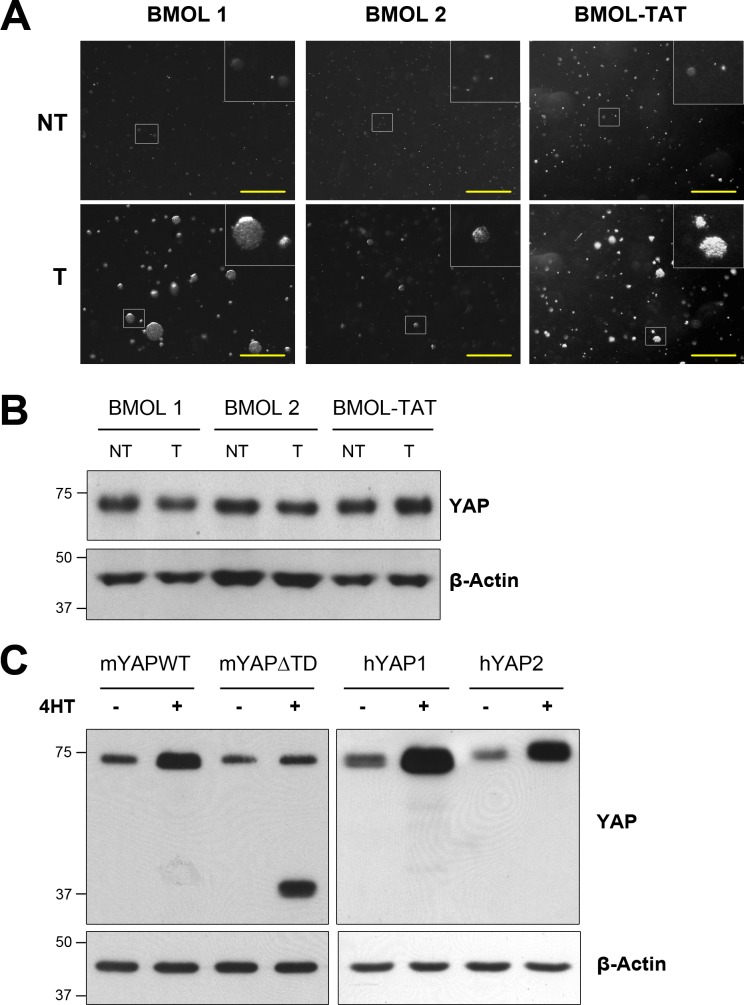
YAP abundance is not increased in transformed LPCs. A) Three independent pairs of non-transformed (NT) and transformed (T) LPCs, BMOL 1, BMOL 2 and BMOL-TAT, were cultured in soft-agar for up to 3 weeks to determine their anchorage-independent growth ability. Scale bars represent 1 mm. B) Cells in (A) were lysed and separated by SDS-PAGE, transferred to membrane and immunoblotted for YAP and the loading control β-actin, as indicated. C) Stably infected NIH3T3 cells were treated with or without 100 nM 4HT for 24 h to induce expression of mYAPWT, mYAP∆TD, hYAP1 or hYAP2 as indicated. Cell lysates were separated by SDS-PAGE, transferred to membrane and immunoblotted for YAP and β-actin as indicated.

We generated three independent pairs of LPC lines in which the transformed cells were derived from the non-transformed cells. First, to confirm their transformed status we cultured each pair of LPCs in soft-agar to determine their capacity for anchorage-independent growth as an indicator of their tumorigenicity. Indeed all transformed LPCs were capable of growing large colonies in soft-agar whereas their non-transformed counterparts were unable to (Fig. 1A). Next we compared YAP abundance in the three pairs of transformed and non-transformed of LPC lines. In contrast with our previous findings [28] we observed that YAP abundance was unchanged in all three transformed LPCs when compared with their non-transformed counterparts (Fig. 1B). This result was unexpected, therefore to confirm the reliability of our data we performed Western blots of lysates of NIH3T3 cells that were induced to express multiple isoforms of mouse and human YAP. The YAP antibody clearly detected full-length mYAP as well as both human YAP1 and YAP2 isoforms (Fig. 1C). The bands for the three wild-type YAP isoforms were the correct size. Furthermore the antibody detected a band of the correct size for mYAP∆TD that lacked the entire C-terminal transactivation domain (TD). YAP was also detected in lysates from uninduced cells and this was most likely due to endogenous YAP and/or leaky expression of the induced construct as observed in the uninduced hYAP samples. This data indicates that the CST antibody is reliable for detecting mouse and human YAP forms by Western blotting. Furthermore this result also indicates that LPCs do not increase YAP abundance following the process of transformation.

**Figure 2:**
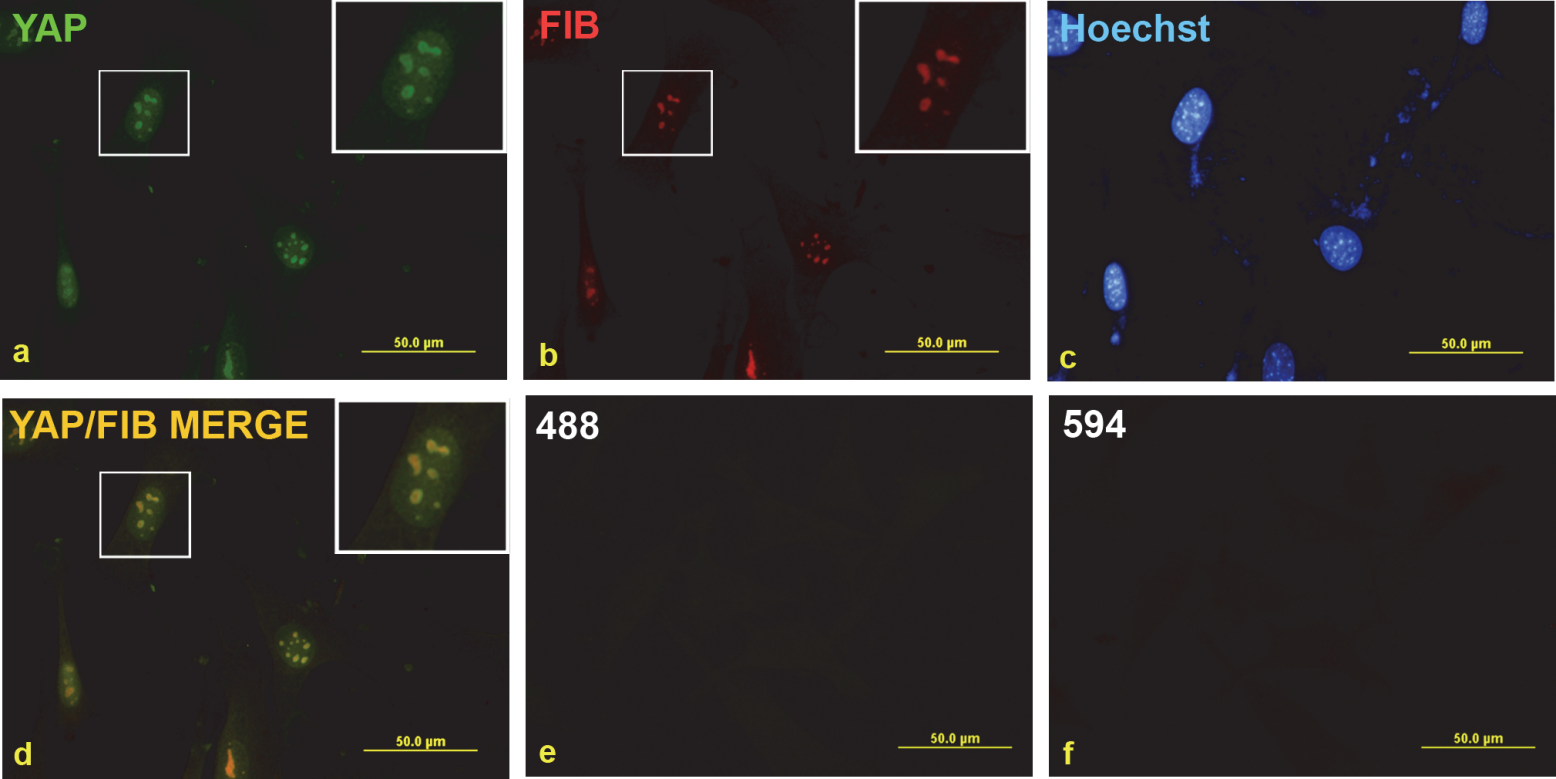
Punctate nuclear YAP staining is present in LPCs and co-localises with the nucleolar marker fibrillarin. Non-transformed BMEL A-EGFP LPCs were grown on coverslips and fixed before being co-stained with YAP (a) and fibrillarin (FIB) (b) antibodies and counterstained with Hoechst stain as shown (c). Overlay of YAP and FIB images in (a) and (b) is shown in (d). Undetectable immunofluorescence was seen with the Alexa Fluor-488 (e) and-594 (f) secondary antibodies alone.

To determine whether YAP sub-cellular localisation is altered following transformation of LPCs we initially stained non-tumorigenic, BMEL A-EGFP LPCs for YAP. Immunofluorescence revealed that YAP was mostly nuclear, with little or no cytoplasmic staining detected (Fig. 2A). Interestingly, a punctate pattern of YAP staining was observed within the cell nuclei. This staining was specific to the YAP antibody since the secondary antibody alone control produced no staining above background (Fig. 2E). We hypothesized that the punctate staining pattern may correspond to the nucleoli within the cells. To confirm this possibility we co-stained for YAP and fibrillarin (FIB), a protein restricted to the dense fibrillar component of the nucleolus [38]. fibrillarin staining revealed a similar pattern to that observed for YAP (Fig. 2B), which superimposed when the two images were overlaid (Fig. 2D). This result confirmed that the punctate YAP staining was localised to the nucleoli.

**Figure 3:**
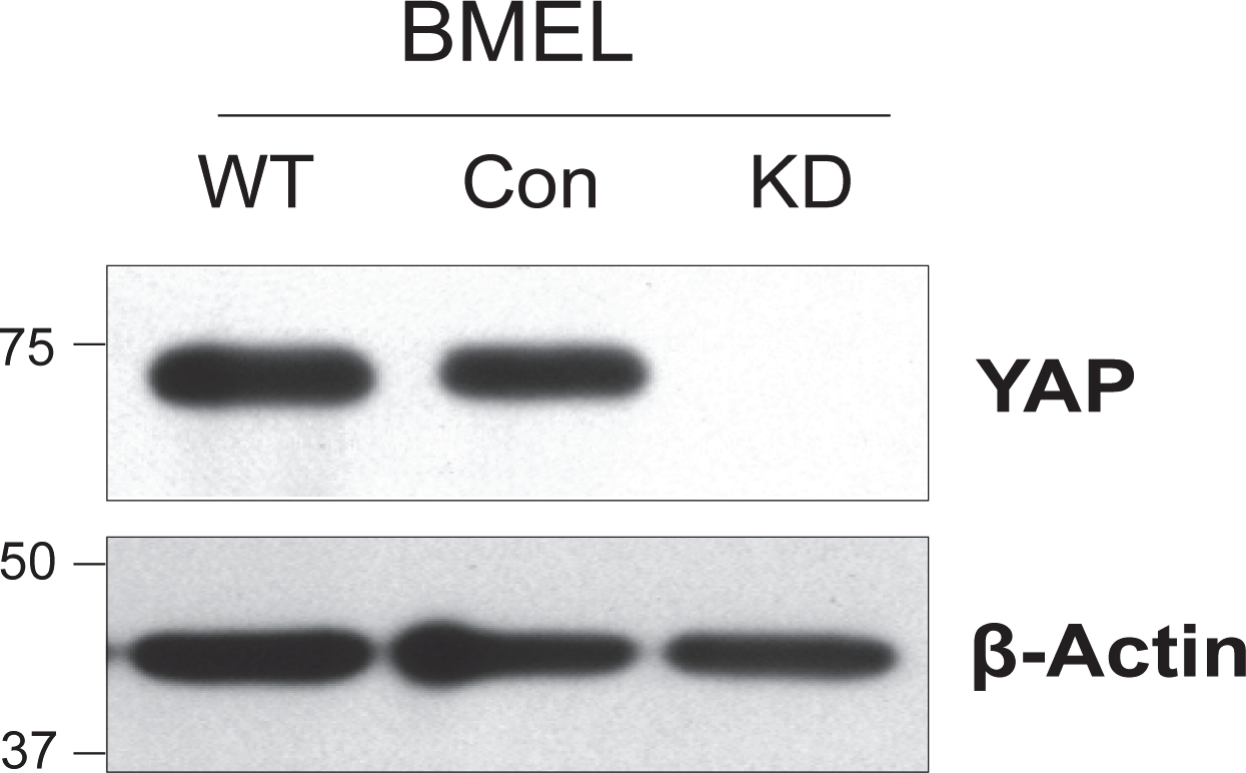
YAP protein is undetectable in shRNA-mediated YAP knockdown LPCs. Wild-type (WT) non-transformed BMEL A-EGFP LPCs (BMEL) were stably infected with lentiviruses bearing control (Con) or YAP-targeting (KD) shRNAs. After selection with puromycin stably infected cells were harvested. Cell lysates were separated by SDS-PAGE, transferred to membrane and immunoblotted for YAP and β-actin as indicated. Size markers are shown in kilodaltons.

To eliminate the possibility that the YAP staining pattern in LPCs was the result of non-specific cross-reactivity we repeated the experiment using BMEL A-EGFP LPCs that had been stably infected with lentivirus bearing YAP-specific or control shRNAs. YAP protein was undetectable in the YAP knockdown (KD) cells, but was easily detected in uninfected cells (WT), or those infected with the non-targeting control (Con) (Fig. 3).

**Figure 4:**
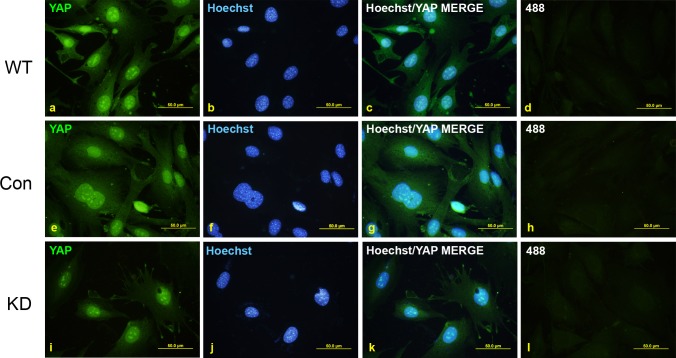
YAP immunofluorescent staining is non-specific in LPCs. Wild-type (WT) non-transformed BMEL A-EGFP cells were stably infected with lentiviruses bearing control (Con) or YAP-targeting (KD) shRNAs. Stably infected cells were grown on coverslips and fixed before being stained with YAP (a, e, i) and counterstained with Hoechst stain (b, f, j). Overlay of YAP and Hoechst images are shown in (c, g, k). Undetectable immunofluorescence was seen with the Alexa Fluor-488 antibody alone (d, h, l).

Despite very efficient YAP knockdown, immunofluorescent staining revealed significant YAP-immunoreactivity remained in the KD cells (Fig. 4). Specifically, the nuclear staining in the KD cells (Fig. 4I) was as intense as for the WT (Fig. 4A) and Con (Fig. 4E) cells, although some cytoplasmic YAP staining in the KD cells was diminished, but not completely absent. Importantly, YAP nucleolar staining in the KD cells was not reduced compared to WT and Con cells (Fig. 4C, G and K). These results indicate that the YAP antibody is reacting non-specifically to the nucleolus in BMEL A-EGFP LPCs.

**Figure 5:**
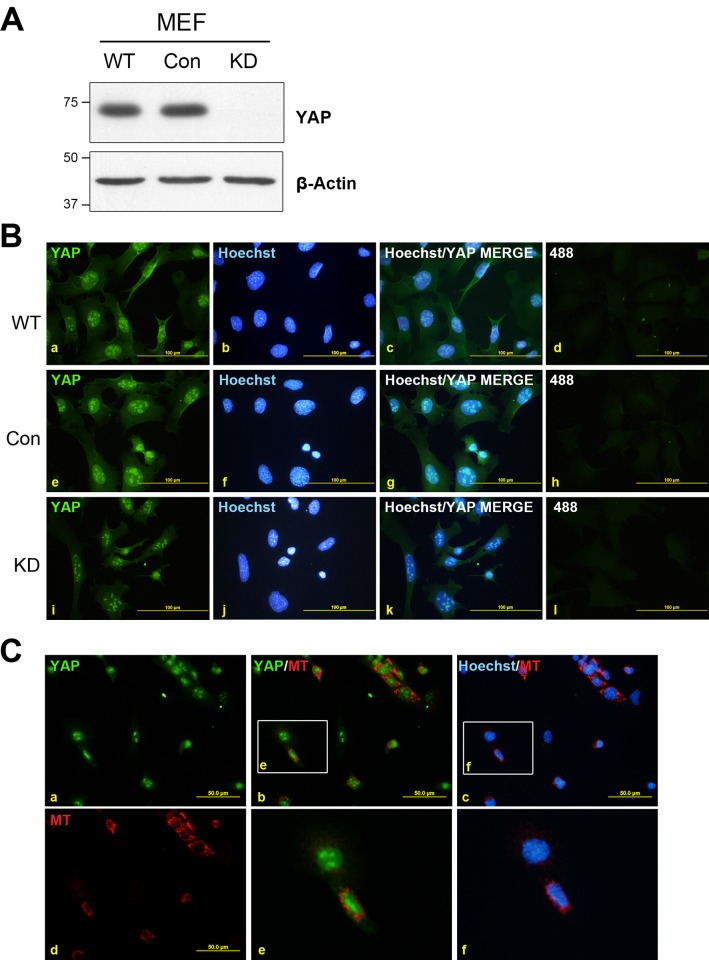
YAP immunofluorescent staining is non-specific in MEFs. Wild-type (WT) MEFs were stably infected with lentivirus harbouring control (Con) or YAP-targeting (KD) shRNAs. **A**) Lysates of stably infected cells were separated by SDS-PAGE, transferred to membrane and immunoblotted for YAP and β-actin as indicated. Size markers are shown in kilodaltons. **B**) Stably infected cells were grown on coverslips and fixed before being stained with YAP (a, e, i) and counterstained with Hoechst stain (b, f, j). Overlay of YAP and Hoechst images are shown in (c, g, k). Immunofluorescence was not detected using Alexa Fluor-488 secondary antibody alone (d, h, l). **C)** Con MEFs were pre-labelled with MT then washed, trypsinized and mixed 1:1 with unlabelled YAP KD MEFs before being replated onto glass coverslips. Cells were allowed to settle for 4 h before being fixed and stained for YAP (a) and counterstained with Hoechst stain. MT was visualised (d) and merged images of YAP/MT (b), and Hoechst/MT (c) are shown, with enlarged insets (boxed regions in b and c) shown in (e) and (f), respectively.

To determine whether the non-specific, nucleolar YAP staining was present in other cell types we repeated these experiments in immortalised wild-type MEFs. YAP was efficiently reduced to undetectable levels by Western blot in cells infected with the YAP-specific shRNA (KD), whereas YAP abundance was not altered by the control shRNA (Con) when compared to the parental (WT) cells (Fig. 5A).

Similar to LPCs, immunofluorescent analysis revealed strong nuclear YAP staining with intense nucleolar immunoreactivity and scant cytoplasmic staining in both WT and Con cells (Fig. 5B, A and E). In contrast, the KD MEFs showed reduced staining within the cytoplasm and nucleus; however, the nucleolar staining was comparable with that seen in the WT and Con cells (Fig. 5BI). To validate this result we then fluorescently labelled Con MEFs with MitoTracker Red (MT) and re-plated these cells onto coverslips together with unlabelled KD MEFs at a ratio of 1:1. 4 h after plating the cells were fixed and stained for YAP. Consistent with the previous experiment a similar nucleolar pattern of staining was observed in all cells (Fig. 5C). Importantly, both the MT-labelled Con shRNA cells (red fluorescent cells) and the YAP shRNA (KD) cells showed a very similar staining pattern (Fig. 5C, panels a, b and e). As expected the presence of MT did not affect the YAP staining pattern (S1 Fig.). Collectively these results clearly indicate that in both murine LPCs and MEFs, the YAP-antibody non-specifically reacts with a target within the nucleus, specifically the nucleoli, when utilised for immunofluorescent approaches.

**Figure 6:**
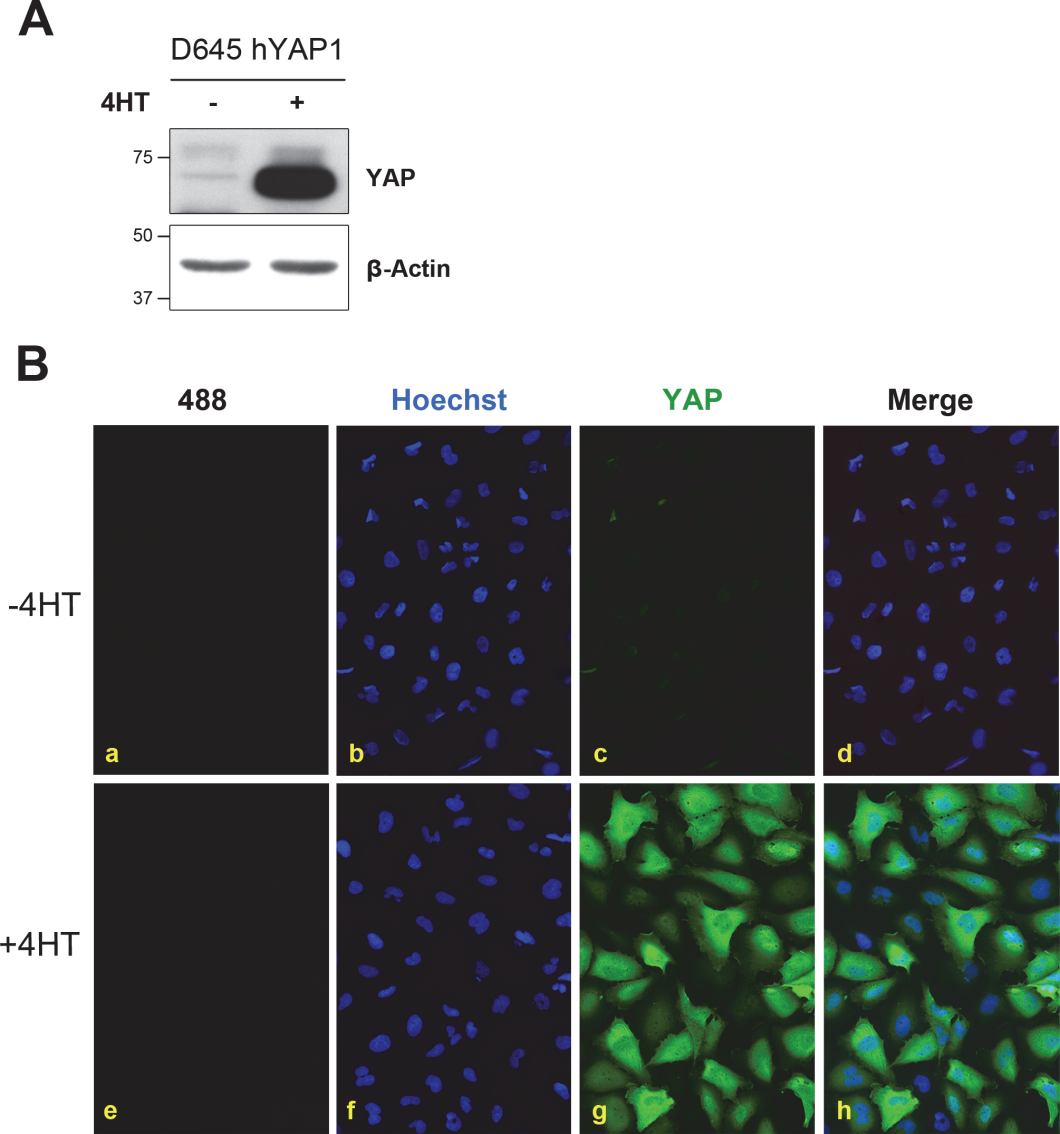
YAP immunofluorescent staining correlates with YAP abundance in D645 cells. Stably infected D645 cells were treated with or without 100 nM 4HT for 24 h to induce hYAP1 expression as indicated. A). Cell lysates were separated by SDS-PAGE, transferred to membrane and immunoblotted for YAP and β-actin, as indicated. B) Cells were grown on coverslips and fixed before being stained with YAP (c, g) and counterstained with Hoechst stain (b, i). Merge of YAP and Hoechst images are shown in (d, h). Undetectable immunofluorescence was seen with the Alexa Fluor-488 antibody alone (a, e).

To determine whether a similar staining pattern was observed for YAP in human cells we stained D645 cells that could be induced to express hYAP1. 4HT treatment robustly induced hYAP1 abundance that was detected by Western blot with minimal background (Fig. 6A). When we stained these cells for YAP we observed diffuse staining throughout the induced and uninduced cells (Fig. 6B). Moreover, the staining intensity was significantly higher in the induced cells compared with the non-induced cells which is consistent with YAP abundance determined by Western blotting (Fig. 6A). Interestingly, the punctate staining pattern for YAP was not observed in these cells suggesting this non-specific staining pattern might be restricted to rodent cells.

## Discussion

Previous studies have reported an increase in YAP abundance and nuclear localisation in tumorigenesis [8, 10–12, 15, 27]. Indeed we previously showed that YAP abundance is similarly increased in tumorigenic LPCs [28], however, in this instance the tumorigenic cells compared were not derived from non-tumorigenic cells but rather arose independently. We now show that in three independent pairs of transformed/non-transformed LPCs YAP abundance is not increased during LPC transformation. In light of our previous finding [28] and that Hippo signaling and YAP itself plays a key role in the liver and liver cancer [8, 10, 13, 15, 27, 39–42] this result was unexpected, however, none of these earlier studies directly examined YAP expression in LPCs nor compared its abundance before and after LPC transformation. Our data indicates that increased YAP abundance is not essential for LPC transformation.

Although YAP abundance did not increase in transformed LPCs, we considered the possibility that YAP activity was up-regulated in these cells via an increase in nuclear abundance. We examined this by immunofluorescence using a widely used antibody. The results presented in this study demonstrate that the CST YAP antibody is unsuitable for immunofluorescent staining of mouse cell lines due to a high level of non-specific immunoreactivity in cells, especially in the nucleoli. This study provides convincing evidence that this antibody cannot be relied upon to determine the sub-cellular localisation of YAP protein *in situ* using immunofluorescent staining due to intense non-specific staining of nucleoli in mouse cells which was not affected by the treatment of cells with YAP-specific shRNA. In contrast the antibody does show specificity in Western blotting applications. Firstly, YAP is not detected by Western blot in shRNA-treated cell lines, and secondly, the antibody can detect several isoforms of the mouse and human YAP proteins. The apparent differences in antibody specificity for these two applications may be explained by the fact that Western blotting detects denatured protein, whereas immunofluorescent staining detects natively folded protein. This may cause non-specific interactions between the antibody and un-related protein(s) that share similar epitopes in their non-denatured states.

A search of the literature identified several publications that have used this particular antibody for immunofluorescent and-histochemical analyses [26, 27, 39]. These include the afore mentioned publication by Oudoff et al. describing Set7-dependent methylation of YAP in MEFs [26], and a highly cited publication describing the affect of NF2 expression on YAP localisation in oval cells (aka LPCs) [39]. Both publications describe an intense punctate sub-nuclear staining pattern in MEFs and oval cells (LPCs), reminiscent of the data presented here. The overall conclusions of these respective studies may still be valid even though they used this antibody to determine YAP’s sub-cellular localisation. However, the present study raises concerns of the validity of the immunofluorescence data and its value in supporting the conclusions drawn on the regulation of YAP. For example, nucleolar staining of YAP was clearly evident in several figures in the paper by Oudoff et al. [26] in which they used the antibody in question to quantify YAP’s sub-cellular localisation without providing any controls, e.g. the use of knockdown cells, to confirm the specificity of their data.

We observed non-specific YAP staining in the nucleolus in two different mouse cell lines, namely MEFs and LPCs, using several staining protocols, including that used for D645 cells, and different batches of antibody. Thus it is highly likely that similar non-specific staining for YAP will be observed in other mouse cell types or tissues. In support of this, mouse liver tissues stained for YAP using immunohistochemistry with the same antibody produced a similar, nucleolar pattern of staining (S2 Fig.). While this is likely to be non-specific it needs to be confirmed by staining similar tissue from YAP knockout mice. However, staining YAP mutant embryos with the same CST YAP antibody produced a similar nucleolar staining pattern which the authors also concluded was non-specific [43]. These observations raise further doubts regarding the reliability of the antibody in immunohistological applications for mechanistic studies of YAP function in murine samples.

In contrast, the staining of human D645 cells with the antibody did not produce the same nucleolar staining pattern for YAP that was evident in mouse cells and tissues. Although we have not formally shown the YAP staining in D645 cells is specific (e.g. by YAP knockdown), this is likely since the intensity of immunofluorescence was in complete accordance with YAP’s abundance as determined by Western blotting of D645 cell extracts with and without hYAP1 over-expression. This suggests that the YAP antibody may be suitable for use with human samples but not with mouse. Irrespective of cell type or tissue being examined the need for controls is paramount for data validation.

The results presented clearly highlight one of the weaknesses of using antibody-based techniques for protein detection *in situ*. Furthermore, they re-emphasize the importance of incorporating adequate controls that validate the antibody using either knockdown or ideally, gene knockout of the target protein, to ensure antibody specificity. Whilst the development of immunofluorescent and immunohistochemical staining techniques, especially in combination with tissue arrays has provided a means for *in situ* protein detection, a thorough appreciation of the weaknesses of these technique is warranted. Often studies are published without the appropriate controls and there is an assumption by the reader that the techniques described are fully controlled and valid. Clearly as we have highlighted in two examples [26, 39] this is not always the case. The utilisation of additional methods to confirm results obtained using immunofluorescence or immunohistochemistry is mandated by the likelihood that staining patterns are subject to a plethora of possibilities of non-specific staining due to the preparation of tissue. These include solutions, fixatives, collection of specimens and treatment of the animal prior to sample collection. Approaches to confirm the validity of immunolocalisation of proteins include sub-cellular fractionation, or expression of exogenously tagged proteins. Unfortunately, these techniques have their own distinct set of limitations, which should be addressed before conclusions can be drawn. For example, exogenous protein overexpression may overwhelm intrinsic regulatory mechanisms including localisation control. Moreover, the addition of protein tags such as GFP may alter protein folding and/or interactions within the cell, resulting in mis-localisation of GFP-fusion proteins. Therefore caution must be exercised in interpreting data obtained from such experiments. A comprehensive analysis using multiple techniques that produce a similar result is more likely to be biologically correct.

We conclude that YAP abundance is not increased in transformed LPCs. Whether nuclear YAP is more abundant in transformed LPCs remains to be determined. These results also serve as a timely reminder of the necessity to perform appropriate controls in sub-cellular localisation experiments, especially when using immunological based techniques. The publication of data without these controls is impossible to interpret with confidence and can lead to entrenched misleading assumptions especially when this data is published in high-impact journals.

## Supporting Information

S1 Fig YAP immunofluorescent staining is non-specific in MEFs. Wild-type (WT) MEFs were stably infected with lentivirus harbouring control (Con) or YAP-targeting (KD) shRNAs. Con MEFs (panels a-e) and YAP KD MEFs (panels f-j) were plated onto coverslips as indicated. Panels k-o: Con MEFs were pre-labelled with MT (Con (MT)) then washed, trypsinized and mixed 1:1 with unlabelled YAP KD MEFs before being replated onto glass coverslips. Cells were allowed to settle for 4 h before being fixed and stained for YAP (a, f, k) and counterstained with Hoechst stain. MT was visualised (b, g, l) and merged images of YAP/MT (c, h, m), and Hoechst/MT (d, i, n) are shown, with enlarged insets (boxed regions in panels m and n) shown in (p) and (q), respectively. Immunofluorescence was not detected using Alexa Fluor-488 secondary antibody alone (e, j, o).
(TIF)

S2 Fig YAP antibody stains nucleoli in mouse liver. The liver from a C57BL/6 mouse that was maintained on a choline-deficient, ethionine-supplemented diet for three weeks was fixed in formalin. Serial sections were stained with or without YAP primary antibody as indicated. Scale bar represents 100 μm.
(TIF)

S1 Methods Immunohistochemistry. Paraffin-embedded formalin fixed liver sections (4 μm) were de-waxed and rehydrated then boiled in antigen retrieval buffer (10 mM Tris, 1 mM EDTA, 0.05% Tween-20, pH 9.0) for 20 min prior to blocking endogenous peroxidases with 3% H_2_O_2_. Sections were blocked with serum-free protein block (DAKO, North Sydney, NSW) for 30 min at room temperature then incubated overnight at 4°C with anti-YAP antibody diluted 1:25 in REAL Antibody Diluent (DAKO). Sections were washed with Tris-buffered saline (TBS) and stained with the LSAB+ kit (DAKO) and visualised using diaminobenzidine (DAB) according to the manufacturer’s instructions. Sections were counterstained with haematoxylin, mounted and viewed with an Olympus CX41. Images were captured with a Nikon DS-Fi1 camera using the 40x objective.
(DOCX)
