# Supplementary figures and images for: Sub-Cellular Localisation Studies May Spuriously Detect the Yes-Associated Protein, YAP, in Nucleoli Leading to Potentially Invalid Conclusions of Its Function
Source: PLoS One. 2015 Feb 6;10(2):e0114813. doi: 10.1371/journal.pone.0114813 (PMC4320119; doi:10.1371/journal.pone.0114813)

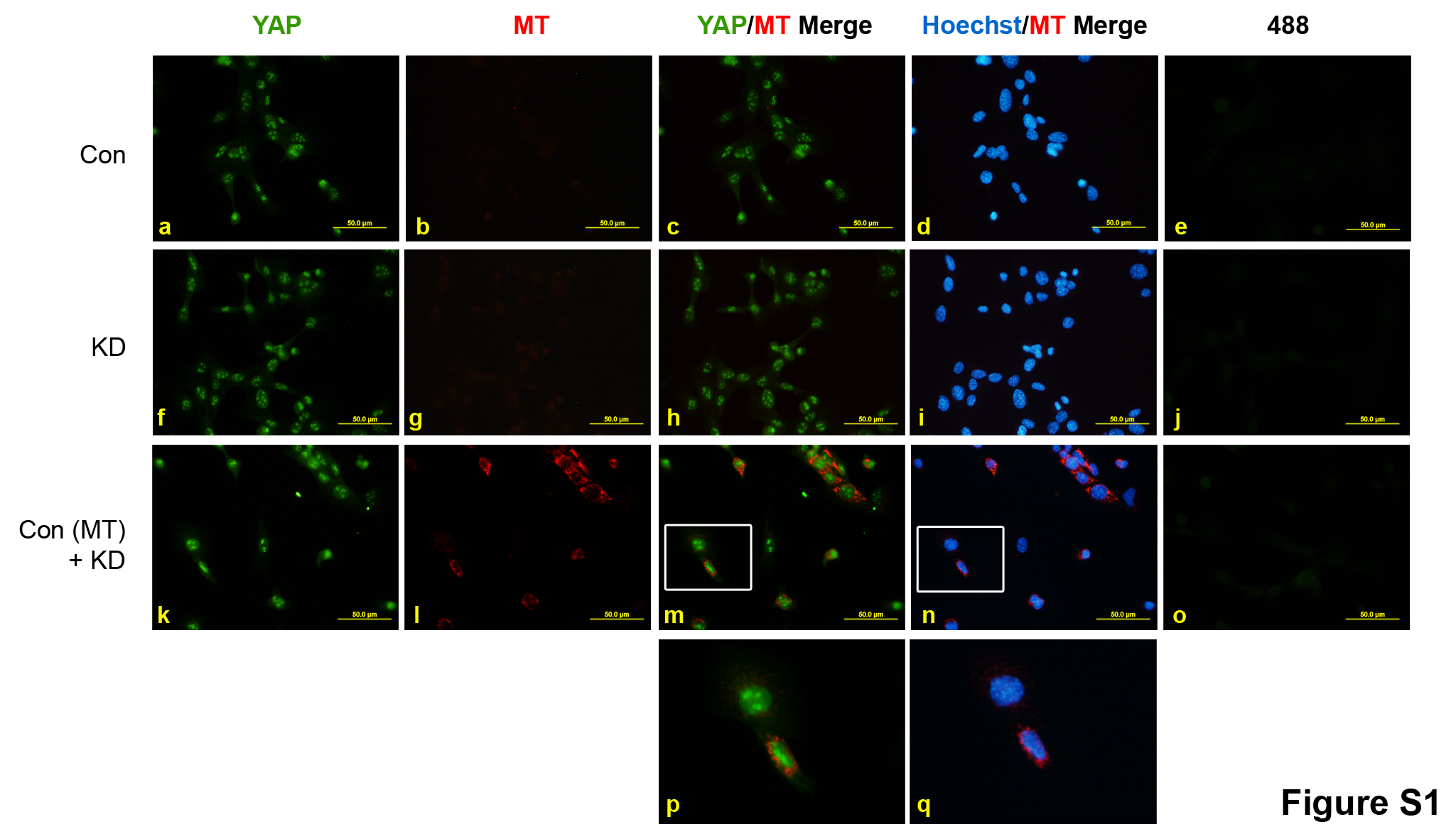

Supplement: S1 Fig — Wild-type (WT) MEFs were stably infected with lentivirus harbouring control (Con) or YAP-targeting (KD) shRNAs. Con MEFs (panels a-e) and YAP KD MEFs (panels f-j) were plated onto coverslips as indicated. Panels k-o: Con MEFs were pre-labelled with MT (Con (MT)) then washed, trypsinized and mixed 1:1 with unlabelled YAP KD MEFs before being replated onto glass coverslips. Cells were allowed to settle for 4 h before being fixed and stained for YAP (a, f, k) and counterstained with Hoechst stain. MT was visualised (b, g, l) and merged images of YAP/MT (c, h, m), and Hoechst/MT (d, i, n) are shown, with enlarged insets (boxed regions in panels m and n) shown in (p) and (q), respectively. Immunofluorescence was not detected using Alexa Fluor-488 secondary antibody alone (e, j, o). (TIF) [file pone.0114813.s001.tif]

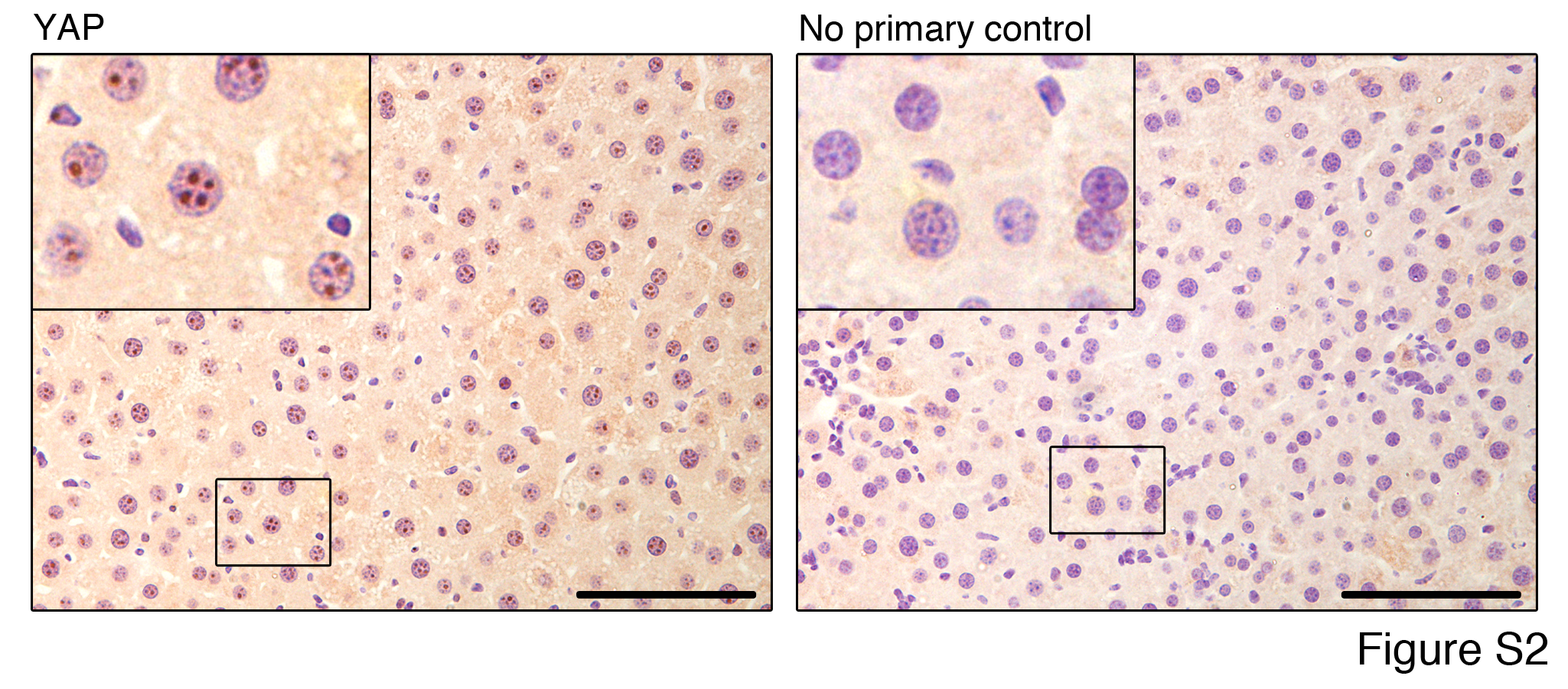

Supplement: S2 Fig — The liver from a C57BL/6 mouse that was maintained on a choline-deficient, ethionine-supplemented diet for three weeks was fixed in formalin. Serial sections were stained with or without YAP primary antibody as indicated. Scale bar represents 100 μm. (TIF) [file pone.0114813.s002.tif]
