# Supplementary material for: Sub-Cellular Localisation Studies May Spuriously Detect the Yes-Associated Protein, YAP, in Nucleoli Leading to Potentially Invalid Conclusions of Its Function
Source: PLoS One. 2015 Feb 6;10(2):e0114813. doi: 10.1371/journal.pone.0114813 (PMC4320119; doi:10.1371/journal.pone.0114813)
Supplement: S1 Methods — Paraffin-embedded formalin fixed liver sections (4 μm) were de-waxed and rehydrated then boiled in antigen retrieval buffer (10 mM Tris, 1 mM EDTA, 0.05% Tween-20, pH 9.0) for 20 min prior to blocking endogenous peroxidases with 3% H2O2. Sections were blocked with serum-free protein block (DAKO, North Sydney, NSW) for 30 min at room temperature then incubated overnight at 4°C with anti-YAP antibody diluted 1:25 in REAL Antibody Diluent (DAKO). Sections were washed with Tris-buffered saline (TBS) and stained with the LSAB+ kit (DAKO) and visualised using diaminobenzidine (DAB) according to the manufacturer’s instructions. Sections were counterstained with haematoxylin, mounted and viewed with an Olympus CX41. Images were captured with a Nikon DS-Fi1 camera using the 40x objective. (DOCX) [file pone.0114813.s003.docx]

**Methods S1.**

**Immunohistochemistry**.

Paraffin-embedded formalin fixed liver sections (4 µm) were de-waxed and rehydrated then boiled in antigen retrieval buffer (10 mM Tris, 1 mM EDTA, 0.05% Tween-20, pH 9.0) for 20 min prior to blocking endogenous peroxidases with 3% H_2_O_2_. Sections were blocked with serum-free protein block (DAKO, North Sydney, NSW) for 30 min at room temperature then incubated overnight at 4°C with anti-YAP antibody diluted 1:25 in REAL^TM^ Antibody Diluent (DAKO). Sections were washed with Tris-buffered saline (TBS) and stained with the LSAB+ kit (DAKO) and visualised using diaminobenzidine (DAB) according to the manufacturer’s instructions. Sections were counterstained with haematoxylin, mounted and viewed with an Olympus CX41. Images were captured with a Nikon DS-Fi1 camera using the 40x objective.
